# Methodology for Constructing Problem Definitions in Bioinformatics

**DOI:** 10.4137/bbi.s706

**Published:** 2008-04-24

**Authors:** Amy M. Hauth, Gertraud Burger

**Affiliations:** Robert Cedergren Center for Bioinformatics and Genomics, Département de Biochimie, Université de Montréal, 2900 Boulevard Edouard-Montpetit, Montréal, Québec, H3T 1J4, Canada

**Keywords:** problem formulation, tool development, guidelines

## Abstract

**Motivation:**

A recurrent criticism is that certain bioinformatics tools do not account for crucial biology and therefore fail answering the targeted biological question. We posit that the single most important reason for such shortcomings is an inaccurate formulation of the computational problem.

**Results:**

Our paper describes how to define a bioinformatics problem so that it captures both the underlying biology and the computational constraints for a particular problem. The proposed model delineates comprehensively the biological problem and conducts an item-by-item bioinformatics transformation resulting in a germane computational problem. This methodology not only facilitates interdisciplinary information flow but also accommodates emerging knowledge and technologies.

## Introduction

A number of recent papers have identified ‘open’ problems in bioinformatics. From a computer science perspective, these problems have been classified broadly into those (i) related to the ‘central dogma’ (i.e. DNA to RNA to protein), (ii) related to data in general and (iii) simulating biological processes (Backofen and Gilbert, 2001). From a life science perspective, open bioinformatics questions are concrete questions, such as, ‘which structural RNAs are encoded in a genome?’ (Eisenberg et al. 2006; Goodman, 2002; Yu et al. 2004). Yet, there is a fundamental difference between the bioinformatics problems described above and the aim of this paper, which proposes a systematic procedure for constructing definitions of such problems.

For the most part, ‘how-to’ practices in bioinformatics address the application of software engineering and database management principles to computational issues. For example, Parker et al. (2003) proposed comprehensive management of information flow for large-scale genome projects through system-wide management of metadata and data dependencies across both biological and computational processes. This led to implementation of numerous integrated systems, commonly referred to as pipelines or workflows (e.g. Garcia Castro et al. 2005). Indeed, these efforts made significant contribution to bioinformatics ‘in-the-large’. Still lacking, however, are how-to-practices for bioinformatics problems ‘in-the-small’.

We propose a methodology for formulating bioinformatics problems by defining the cognate life and computational problems in an explicit and integrated fashion. The goal of this methodology is to guide development of bioinformatics tools that account for critical biology. Our work is much different from what is typically published in the field of bioinformatics. We focus on methods for formulating a problem rather then for solving an already formulated problem.

## Methodology

Our procedure has three components: a biological model, a bioinformatics transformation and a computational model (Table 1). The biological model specifies a question of interest and defines the biological problem in a way that captures the breadth of the phenomenon. The bioinformatics transformation translates biological features and criteria into a set of computational rules, which, as a whole, circumscribe the biological problem. Finally, the computational model reformulates the problem mathematically by incorporating rules derived in the transformation and describes a computational approach to the initial biological question.

As an example of our methodology, we pose the biological question ‘which tRNAs are encoded in a genome?’ Information on tRNAs is available in the supplemental materials and in the literature (e.g. Marck and Grosjean, 2002; Paule and White, 2000; Sprinzl et al. 1998).

### Biological model

A biological model consists of a concise ‘biological question’ and a comprehensive description of the ‘biological knowledge.’ Both portions are critical to the entire bioinformatics model. The biological question is usually straightforward; for instance, the case example used here seeks to detect tRNA genes (i.e. a known phenomenon) in genomic sequences. In contrast, formulating the knowledge portion is more difficult requiring a comprehensive, taxonomically broad review of the life science literature and other available resources. As we detail below, the knowledge portion has three elements: (i) an abstract, ‘global’ definition of the phenomenon together with a textual exposé of observed scenarios, (ii) a comprehensive dataset of observed instances, and (iii) a description of yet unobserved, conceivable scenarios. Obviously, the state of knowledge about a particular biological problem determines how the question is formulated and how the biological phenomenon is described (see for example the early work on tRNA sequence and structure (Holley et al. 1965; Levitt, 1969)).

A sample biological model for tRNA gene identification is available in the supplemental Table S1. For the sake of simplicity, the model does not include more advanced criteria such as minimum free energy.

#### Definition and observed variation of a biological phenomenon

**Figure S1:**
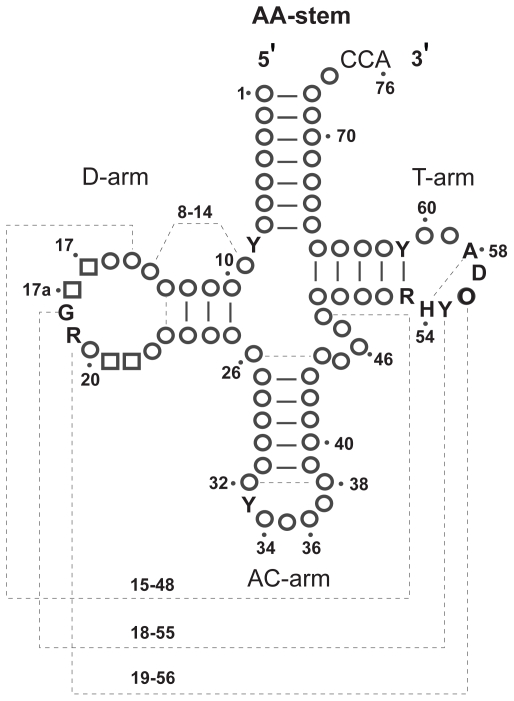
Canonical schematic of the tRNA cloverleaf secondary structure (Marck and Grosjean, 2002).

One first provides a brief, abstract ‘textbook’ definition that views the phenomenon in a larger biological context, together with a typical scenario. Then, one should describe the extent and frequency of biological diversity, both within an organism and across taxa indicating both significant and minor differences. For tRNA gene identification, this may read as follows:

Transfer RNA molecules have two significantly different types of secondary structures, the cloverleaf and the two-arm. The more common structure consists of four stems (or three-arm) in the form of a cloverleaf (Fig. S1). Yet, an unusual three-stem (or two-arm) type is common in certain animal lineages (Okimoto and Wolstenholme, 1990, Fig. S2). Notably, both types of tRNAs fold into a similar L-shaped tertiary structure (Fig. S3). … A minor difference observed is the size of the D-arm loop (D-loop), which typically is 8 nt long but can be up to 10 nt long.

The description of the phenomenon should be comprehensive yet constrained to relevant features. For example, if the question involves identification of tRNA genes in genomic sequences, introns are relevant, but not so if the question solely addresses tRNA secondary structure. Similarly, mapping of a codon to an amino acid (the genetic code) is irrelevant for defining tRNA secondary structures, but relevant for identifying tRNA function.

#### Compilation of a comprehensive dataset

Concrete instances of a phenomenon make a description explicit and tangible. A compilation should span the breadth of taxonomic diversity and should contain a sampling of frequent occurrences as well as all known instances of rare and unique ones. As we discuss later, such a collection will be crucial for benchmarking bioinformatics tools.

#### Description of conceivable scenarios

Life scientists continually uncover novel occurrences of a given biological phenomenon, and not infrequently, these novelties fall within the expected range of diversity. To accommodate future discoveries within the framework of the biological model, one may include knowledge about biological structures and mechanisms that enable extrapolation of unobserved scenarios. Knowledge may be inferred from the same system, or from other systems or even other disciplines. For example, we know that a discontiguous molecule can assume the same structure or function as one that is contiguous. Thus, we can extrapolate that a tRNA gene could be encoded by multiple pieces, which are transcribed independently and join post-transcriptionally to form a functional structure. In fact, RNA ‘in pieces’ have been documented for ribosomal RNA (rRNA) of mitochondria from several eukaryotic groups and bacteria (Evguenieva-Hackenberg, 2005 and references therein) and rare examples for discontiguous tRNA genes have been reported as well (Randau et al. 2005; Soma et al. 2007).

A comprehensive biological model, as illustrated above, leads directly into the biological criteria of the next component, the bioinformatics transformation phase.

### Bioinformatics transformation

This component converts a biological description into a set of mathematical formulas. The translation process involves conversion of the biological knowledge description into biological criteria and transformation of these criteria into computational rules. A sample bioinformatics transformation for tRNA gene searches is available in the supplemental Table S2; note that all sample criteria below are excerpts from this table.

#### Biological criteria

This first step converts the biological description into simple, concise verbal statements, termed biological criteria (BCs). A separate criterion is formulated for each characteristic of a feature, such as the length variation allowed for the stem of the D-arm (D-stem) of a tRNA, e.g.

The D-stem length is 3 or 4 nt.

In addition to this list of criteria, it is useful to group related statements (e.g. particular features or major variants) in order to add meaning and to aid organization. See Table S2 (section BC) for criteria for searching tRNA genes in genomic sequences.

After conversion to BCs, check statements for ambiguity. For instance, the criterion above indicates that the D-stem in tRNAs can vary in length. Yet, this statement is ambiguous since it is uncertain what kind of pairings make up a stem—only Watson-Crick pairings or also interactions such as G-U and G-G. To clarify this ambiguity, a new criterion that defines permissible pairings must be added:

Allowable nucleotide pairs: A-U, C-G and G-U.

More generally, clarification of the intended biological meaning may involve adding new or enhancing already formulated criteria.

##### Computational rules

This step is the most crucial one of the bioinformatics transformation. Here, the BCs described above are converted into mathematical formulas, called computational rules (CRs). Generally, a single BC leads directly to a single CR, such as the one-to-one mappings exemplified by nucleotide pairings:

Valid nucleotide pairs (DNA) = {A-T, T-A, C-G, G-C, G-T, T-G}

and D-stem length variation:

3<= | D-stem | <= 4

However, occasionally it may be necessary to combine several BCs to form a single CR (a many-to-one mapping). For example, a CR describing the length of a D-arm combines the following five BCs:

The D-arm forms a hairpin closed by a stem (pos. 10 to 25).

The D-stem length is 3 or 4 nt.

The D-stem pairing positions: 10–25, 11–24, 12–23 and 13–22. Note: if 13–22 do not pair, the numbering remains as though they are in the stem.

The D-loop length is 8 to 10 nt. If positions 13 and 22 do not pair, it increases to 7 to 11 nt.

The D-loop positions: 14, 15, 16, 17, 17a, 18, 19, 20, 20a, 20b, 21. Optional positions: 17a, 20a and 20b.

into a single CR:

16<= | D-arm | <= 18.

Alternatively, a single BC may be utilized by several CR (a one-to-many mapping), such as permissibility of stem bulges used by each of the four stems (see Table S2; BC 3.1 is used by CRs 1.5, 1.6 and 1.9).

An important feature of the conversion step is the explicit mapping of BC to CR, which acts as a conduit that shuttles knowledge through the model. As laid out in the discussion, this mapping facilitates two important tasks: updating a bioinformatics model to accommodate new biological discoveries and assessing the biological capabilities of tools.

Some of the CRs represent the core of the problem, whereas others represent peripheral details. It is important to identify and mark as ‘key’ those features that are essential for the bioinformatics model. For the tRNA example, the basic cloverleaf and two-arm structures are critical features of the *biological phenomenon*. The search for intron-less tRNA genes may be central to a particular *biological question*. Finally, the T-arm and D-arm consensus sequences are essential for effective *computational analysis*. Rules marked as crucial receive special attention, not only during construction of the computational model but also later, during software development when infeasibility causes modification of the problem and removal of required rules (requirements).

### Computational model

The third phase of defining bioinformatics problems consists of reformulating the biological problem into a pure computational one. Unlike the two previous components, this phase does not devise a specific procedure, as techniques for defining a computational problem (model) are well established. Instead, we focus on *what* to include in such a definition.

First, a global problem definition should re-state the biological question as a computational problem. For example,

Problem: Given a DNA sequence, S, locate all genes, G, capable of forming a functional tRNA structure.

Second, we define a set of smaller problems (problem-set) that together describe a general approach satisfying the global problem. Each (smaller) problem should define a specific task and should state explicitly the CRs that apply to this particular problem. In addition, even the most trivial assumptions required by a problem should be stated explicitly in the definition. For example, assuming a four-nucleotide alphabet (for RNA sequences) lends itself to a highly efficient, bit-based computational approach. If the alphabet size would increase, this approach would be less effective and the problem itself may require revision. Third, each CR must be stated as a requirement for at least one (smaller) problem within a set. More challenging is determining all problems that rely on a given CR.

Obviously, more than one computational approach can address the same global problem; hence, alternative problem-sets can be formulated. For example, identifying tRNA genes using a machine learning approach may subdivide the problem in a manner that differs greatly from a purely deterministic, algorithmic approach. We recommend specification of all these alternatives as they facilitate development and comparison of software that use alternative computational techniques, be it hidden Markov models (Rabiner, 1989), Bayesian networks (Pearl, 1988), or rule-based systems (e.g. (Snyder and Stormo, 1993)).

## Discussion

A bioinformatics model that is constructed according to the proposed methodology captures a problem in its entirety. This is achieved by specifying three separate yet inter-connected modules: a comprehensive biological description of the problem, a computational definition of the problem and an explicit transformation from one to the other.

Models constructed with our procedure provide a solid foundation for development, testing and comparison of analytical bioinformatics tools. Software development can focus fully on efficiency because the model ensures correct translation of the biology into a computational problem. Tools can be tested more easily, since comprehensive positive test data are readily available in the biological model. Once tools are available based on the same model, they can be compared to determine adherence to the model, performance against a benchmark dataset as well as time and space efficiency; all of these facilitate selection of the ‘best’ tool for a given analytical task.

### Information management and software engineering

Systematic procedures and information management principles are not new in bioinformatics. Informatics-leaning bioinformaticians have been applying these strategies for many years through explicit management of rules (‘requirements’), tracking of relationships between rules as well as data (‘dependencies’) and clear definitions of the extent of the problem (‘scope’). Currently though, such principles are applied predominantly to the informatics realm of bioinformatics rather than to bioinformatics as a whole, spanning both the informatics and biology realms.

### Interdisciplinary communication

For any interdisciplinary science, effective communication is a challenge. Bioinformatics has to deal with differences inherent to the life and computational sciences in terms of basic notions, ways of reasoning and scientific language. These differences present a substantial barrier to both comprehending and explaining ideas. Less obvious is a fundamental difference in conveying information. For instance, life science aims at extracting common patterns from the full breadth of natural diversity. In contrast, computer science aims at bounding a problem by defining assumptions, rules and constraints. Consequently, each side reduces the breadth of the problem through either generalizations or bounds. These reductions must be communicated.

The advantage of our methodology is that it facilitates interdisciplinary communication. First, the scientific language of a particular discipline is used to ensure accurate biological and computational models while translation from one model into the other occurs in a separate step. This provides explicit connections between the two models, connections that link specific biological criteria with specific computational constraints. As a consequence, tracing back a criterion/constraint from one model to the other becomes an easy task.

### Changes in knowledge and technology

Biological sciences are about discovering new features of Life. Therefore, bioinformatics tools and resources need to incorporate new knowledge continually. For example, an early definition of *gene* regards it as a contiguous region on a chromosome that specifies an RNA or protein product. The subsequent discovery of alternative splicing and trans-splicing impacted fundamentally the assumptions underlying gene-finding tools.

Our methodology anticipates both incorporation of new knowledge and application of new technology. Advances in the life sciences can be accommodated because the model records relevant biological facts in a systematic fashion and specifies how they are interconnected with computational rules. Similarly, new computational technology can be accommodated because the model defines the scope and requirements for tool development. At most, a new problem-set needs to be added to the computational model. Construction of this new problem-set is simplified substantially by the computational rules contained in the transformation module.

### Putting this proposal into practice

To allow cooperative model formulation and tool development, models should be openly available (pref. web-accessible). To accommodate new science, they should be easily expandable (e.g. managed in a database). Finally, new models need to reuse components of existing models (e.g. the description and the translated rules for nucleotide pairing). This not only reduces scientific effort and improves speed of model construction but also retains a consistent scientific representation across different bioinformatics models.

## Conclusion

Bioinformatics needs standards and methodologies that span its entire breadth from biology to informatics. Establishment of systematic procedures is necessary to transform the ‘art’ of defining bioinformatics problems into a science.

## Supplementary Material

### Computational model—problematic simplifications

Certain computational practices imprecisely simplify bioinformatics problems. For example, an algorithm used to solve a previous problem is re-used to address a new problem, or alternatively, a problem is well-formulated but does not address known exceptions. More generally, three levels of simplification can negatively affect bioinformatics analysis and therefore should be utilized with extra caution.

### Exclusion of computational rules (CR)

One obvious type of simplification is to disregard certain biological scenarios. For example, when searching tRNA genes, one may ignore all rules associated with the two-arm tRNA structure and focus only on the cloverleaf shape. Omission of CR for biological exceptions can significantly simplify both the global task and individual problems in the problem-set, but it also can significantly change the analytical capabilities of the tool.

### Partial omission of CR

Certain simplifications represent an oversight during construction of the computational model. For instance, an individual problem in a problem-set may locate the tRNA anticodon (AC) arm without accounting for introns in the stem, while another problem may explicitly look for introns. However, if intron search is performed after AC-stem identification, then genes with an intron in the AC-stem will not be located. Here, intron analysis occurs in a separate (smaller) problem and yet, the scenario where introns occur in the AC-stem is not analyzed correctly. Thus to resolve this case, one would need either to conduct intron search prior to stem identification or to add a CR for introns to the stem identification step. This example illustrates that some CR may be essential to more than one individual problem and one must ensure that each CR serves as a criterion for *every* relevant problem.

### Inaccurate computational assumptions

Inaccurate, implicit assumptions can change the technical nature of a computational problem in the same manner as omitting CR. For example, suppose that stem-permissible base pairings were not specified as a rule during the bioinformatics transformation phase (i.e. Table S2: BC 3.2). An inaccurate assumption could be that only Watson-Crick (WC) nucleotide interactions are valid pairings in a stem. Then, finding two pairing sequences capable of forming a stem is equivalent to identifying inverted repeats since inverted repeats can pair to form stems. Yet, stems containing non-WC interactions will not be identified. To uncover such a fundamental error requires a good understanding of both the computational and biological implications.

### Impact

Explicit or implicit omission of CR can lead to such a principal change that the biological question is no longer addressed appropriately. In the previous example, without explicit allowance of non-WC pairs, one assumes that stems contain only WC pairs. This assumption creates a trivial computational problem where the sequence on one side of the stem can pair with only *one* other sequence, a problem solved by many existing algorithms (e.g. string matching algorithms). On the other hand, if non-WC pairs are permissible then the sequence on one side has numerous potential sequence pairings for the other side, *F(n)*. This is stated mathematically as follows.

Given a sequence S of length n, the number of sequences having permissible pairings at every position in S is

$$F(n)=p^{w}\times q^{x}\times r^{y}\times s^{z}$$

where w is the number of positions in S having an A, x is the number having C, y is the number having G, z is the number having T/U and w + x + y + x = n and where p is the number of permissible pairing interactions with nucleotide A, q is the number with C, r is the number with G, s is the number with T/U.

Exclusive use of WC pairs means that *p* through *s* has a value of one (a one-to-one mapping of A with T/U and C with G) and that the number of sequences, *F*(*n*), pairing to *S* is one. Further interactions in addition to WC pairs mean that one or more of *p* through *s* will have a value greater than one. For instance, if G can interact with C, G and U then *r* has a value of three and the number of matching sequences increases at an exponential rate, *r^y^*.

Obviously, a minor simplification can have a devastating effect on the analytical results. Less obvious, simplifying also can lead to poor selection of an algorithm, one which cannot easily be extended to accommodate omitted CR. Thus, details which may seem minor to life scientists are crucial to framing the computational problem. This is clearly the case with specification of stem-permissible base pairings where a WC-only assumption not only increases the rate of false negatives but also leads to choosing an “efficient” algorithm which is incapable of handling non-WC pairings.

By this example, it may seem that all simplifications have an adverse effect, but this is not the case. Rather, one should be aware of the pitfalls inherent in common computational practices and take appropriate precautions during development and use of the computational model. In conclusion, it is important that the approach presented in the computational model spells out the algorithmic tasks in a manner that not only satisfies the computational requirements but also correctly answers the original biological question.

## Excerpts from a Bioinformatics Model for tRNA Gene Identification

**Table S1:** Sample of biological model for tRNA gene identification model.

Biological model
Question: Which tRNAs are encoded in a genome?
Relevant knowledge on tRNAs (description of gene, gene product and function) 1. Brief definition “Transfer RNA (tRNA) … is a small RNA molecule (70–90 nucleotides). The tRNAs, by binding at one end to a specific codon in the mRNA and at their other end to the amino acid specified by that codon, enable amino acids to line up according to the sequence of nucleotides in the mRNA. Each tRNA is designed to carry only one of the 20 amino acids …. Each of the 20 amino acids has at least one type of tRNA assigned to it, and most have several tRNAs. Before an amino acid is incorporated into a protein chain, it is attached by its carboxyl end to the 3’end of … a tRNA containing the correct **anticodon**—the sequence of three nucleotides that is complementary to the three-nucleotide **codon** that specifies that amino acid on an mRNA molecule. Codon-anticodon pairings enable each amino acid to be inserted into a growing protein chain according to the dictates of the sequence of nucleotides in the mRNA, thereby allowing the genetic code to be used to translate nucleotide sequences into protein sequences.” (Alberts et al. 1994). 2. Description 2.1 Observed tRNAs (gene product) and genes 2.1.1 tRNA structure “tRNAs can form the loops and base-paired stems of a cloverleaf structure, and all are thought to fold further to adopt the L-shaped conformation” (Alberts et al. 1994). The cloverleaf structure is composed of three arms (D, anticodon (AC) and T), a highly variable (V) loop between the AC- and T-arms, enclosed by the aminoacyl (AA) stem (Fig. 1). Generally, the D-stem forms four base pairings but a stem of three is possible. Likewise, the D-loop is typically 8 nt long but may expand to 9 or 10 nt. Overlapping the D-arm is one of two promoters recognized by transcription factor TFIIIC and having a conserved sequence of 5′-GTGGCNNAGT-3′ (Marck and Grosjean, 2002; Paule and White, 2000). A major alternative to the cloverleaf structure is composed of two instead of three arms, lacking either the D- or the T-arm (Fig. 2). Often, the V-loop expands and establishes extra stabilizing interactions. Such tRNAs lacking entire domains have been documented in certain animal lineages (Sprinzl et al. 1998). In Archea, the ‘strictly invariant’ nucleotide U at position 8 is replaced by a C (Marck and Grosjean, 2002). ***etc.*** Available general resource—http://www.uni-bayreuth.de/departments/biochemie/trna. 2.1.2 tRNA genes The sequence of the tRNA (gene product) differs from that of its gene. The transcribed sequence of the gene is subjected to various processes, which effectively changes the sequence of the gene. Most common are post-transcriptional nucleotide modifications. For example, the T-loop contains the modified base pseudouridine (phi), which is encoded in the gene as T. In addition, the CCA tail at the 3′ end of tRNAs is added post-transcriptionally. Less common are changes incurred by RNA editing by which nucleotides are replaced, inserted or deleted. For example, mis-pairings in the AA-arm portion of the gene between 1–72, 2–71, and 3–70 are corrected post-transcriptionally by RNA editing (Bullerwell and Gray, 2005, and references therein). 2.2 Sample instances Below is a list of sequences representative of tRNA genes. This list is sufficiently broad to serve as a benchmark that measures the effectiveness of tRNA identification software. Journal references are provided where appropriate. GenBank Acc. No. DQ256197, positions 78–145 and references therein. ***etc.*** A compilation of tRNA genes identified by tRNAscan-SE (Lowe and Eddy, 1997) in complete or nearly complete genomes is available at http://lowelab.ucsc.edu/GtRNAdb. Additional tRNA resources are available at http://www.uni-bayreuth.de/departments/biochemie/trna. 2.3 Conceivable genes The unifying structure is the L-shaped tertiary structure required to perform its translational function (Fig. 3). Nucleotides are also important for processing amino-acylation, binding of initiation and elongation factors, etc. The constraints on the shape are …. It is conceivable that the gene is encoded by multiple gene pieces that are transcribed independently, similar to ribosomal RNA (Evguenieva-Hackenberg, 2005).

**Table S2:** Sample of bioinformatics transformation for tRNA gene identification model.

Bioinformatics transformation
Biological criteria (BC) 1. Cloverleaf variant 1.1. This variant is composed of three arms, a stem and a highly variable “bulge” loop: the D-arm(positions 10..25), the anticodon (AC) arm (pos. 27..43), the variable (V) loop (pos. 44..48) and the T-arm (pos. 49..65) enclosed by the acceptor (AA) stem (pos. 1..7 and 66..72). More details in Fig. 1. 1.2. Stems represent the major source of tertiary structure stabilization. Individual nucleotide interactions between loop regions provide some additional stabilization. 2. Two-arm variant 2.1. This variant is composed of two arms, a stem and a highly variable “bulge” loop. The order is similar to the cloverleaf variant, except either the D-arm or the T-arm is absent. See Fig. 2. 2.2. Both stems and individual nucleotide interactions between loops stabilize the tertiary structure. Compensation for the missing stem is provided by a larger V-loop and an increase in non-stem, nucleotide interactions. 3. Stems 3.1. Bulges of one or two nucleotides may occur in a stem 3.2. Allowable nucleotide pairs: A-U, C-G and G-U 4. D-arm 4.1. The D-arm forms a hairpin closed by a stem (pos. 10 to 25). 4.2. The D-stem length is 3 or 4 nt. 4.3. The D-stem pairing positions: 10–25, 11–24, 12–23 and 13–22. Note: if 13–22 do not pair, the numbering remains as though they are in the stem. 4.4. The D-loop length is 8 to 11 nt. If positions 13 and 22 do not pair, it increases to 10 to 13 nt. 4.5. The D-loop positions: 14,15,16,17,17a,18,19,20,20a,20b,21. Optional positions: 17a, 20a and 20b. 4.6. Detailed nucleotide and base-pairing distributions are available (see Tables 1 and 2 in Ref. Marck and Grosjean, 2002) 5. Conserved sequences 5.1. In eukaryotes, the conserved sequence, 5′-GTGGCNNAGT-3′, is found at position 8 (Sharp et al. 1981).
Computational rules (CR) 1. tRNA genes: grammar (partial) 1.1. <tRNA gene>::= <AA-stem begin><ss-loop><three-arm> (BC 1.1, 2.1) <ss-loop><AA-stem end> | <AA-stem begin> <ss-loop><two-arm><ss-loop><AA-stem end> 1.2. <three-arm>::= <D-arm><ss-loop><AC-arm><V-loop>< T-arm> (BC 1.1) 1.3. <two-arm>::= <D-arm><ss-loop><AC-arm><V-loop>| (BC 2.1) <AC-arm><V-loop>< T-arm> 1.4. <D-arm>::= <stem-loop> (BC 4.2) 1.5. <stem-loop>::= <stem begin> <ss-loop> <stem end> | (BC 3.1) <stem begin> <stem-loop-with-bulge> <stem end> 1.6. <stem-loop-with-bulge>::= <ss-bulge> <stem-loop> <ss-bulge> | (BC 3.1) <ss-bulge> <stem-loop> | <stem-loop> <ss-bulge> 1.7. <ss-loop>::= <sequence> general 1.8. <sequence>::= <nucleotide>* general 1.9. <ss-bulge>::= <nucleotide> | <dinucleotide> (BC 3.1) 1.10. <dinucleotide>::= <nucleotide><nucleotide> general 1.11. <nucleotide>::= A | C | G | T general 2. Stems 2.1. *Valid nucleotide pairs(DNA)* = {A-T, T-A, C-G, G-C, G-T, T-G} (BC 3.2) 3. D-arm 3.1. 16 <= | *D-arm* | <= 19 (BC 4.1–4.5) 3.2. 3 <= | *D*-*stem* | <= 4 (BC 4.2) 3.3. 8 <= | *D-loop* | <= 13 (BC 4.3, 4.4) 4. Conserved sequence near D-arm 4.1. D conserved sequence pattern: GTGGCNNAGT (BC 5.1) 4.2. 2 <= | First position of D-stem relative to first position of D-consensus pattern| <= 3 (BC 5.1, 4.2)

**Table S3:** Sample of computational model for tRNA gene identification model.

Computational model
Global problem
*Given a DNA sequence, S, of length n over the alphabet D* = *{A,C,G,T} and the accompanying list of CR, determine the location, L, of all tRNA genes and for each, indicate their putative secondary structure.*
Partial problem-set
1. Identify conserved sequences
*Given a DNA sequence, S, of length n and a set of conserved sequence patterns, P, find all locations, L_p_, in S for each conserved pattern, p, in P.*
*S*, DNA sequence provided by the user
*P*, set of conserved sequence patterns:
*Conserved sequence near D-arm, p_D_: CR 3.4*
*Conserved sequence near T-arm, p_T_: unspecified*
2. Identify candidate arms (form hairpins for stems overlapping conserved patterns)
*Given S, L_p_, and for each p in P, both S_p_, the stem constraints for the arm that overlaps p and D_ps_, the location of the stem relative to p, form stems for each l in L_p_as positioned by D_ps_, and as constrained by S_p_. Each valid stem defines a candidate arm.*
*S_A_*, stem constraints for the arm, *A*, that overlaps the pattern, *p*
*D-stem, s_D_, constraints for D-arm, A_D_: CR 2.1, 3.1, 3.2*
*T-stem, s_T_, constraints for T-arm, A_T_: CR 2.1, unspecified*
*D_ps_*, position of the stem, *s*, relative to the pattern, *p*
*Position of p_D_relative to D-stem, s_D_: CR 3.5*
*Position of p_T_relative to T-stem, s_T_: unspecified*

**Table S4:** Introns in tRNA genes and the effect on the bioinformatics model.

Bioinformatics model
Relevant Knowledge
Addition to tRNA gene description
Introns …
**Bioinformatics transformation**
New biological criteria (NBC) 6. Introns 6.1. Infrequent occurrence in tRNA genes 6.2. Location of introns 6.2.1. Most frequent in loops 6.2.1.1. Most prevalent in V-loop between AC-arm and T-arm 6.2.2. Typically, infrequent in stems 6.2.2.1. In Archaea, introns are predominately present in stems 6.3. Group I introns 6.3.1. Observed occurrences vary from 140 to over 2,000 nt long 6.4. Group II introns (Bonen and Vogel 2001) 6.4.1. Intron structure contains six domains: I-IV 6.4.2. Typical length is 600 to 2,500 nt long. Smallest is 389 nt long. Largest is 3,400 nt long. 6.4.3. Large introns most often contain an ORF, typically in the loop of domain IV. 6.4.4. Domain V sequence: 5′-RAGCYNNRURMrNNrAAANNYKYayGYNNRGUUY-3′
New computational rules (NCR) 5. tRNA genes: additional grammar for introns 5.1. <ss-loop>::= <sequence> | <sequence-or-empty> (NBC 6.2.1, Replaces CR 1.7) <sequence-with-intron> 5.2. <sequence-or-empty>::= <sequence> | ɛ (empty set) general 5.3. <sequence-with-intron>::= <intron> | <intron> <ss-loop> (NBC 6.2.1) 5.4. <ss-bulge>::= <nucleotide> | <dinucleotide>| (NBC 6.2.2, Replaces CR 1.9) <intron-in-bulge> 5.5. <intron-in-bulge>::= <intron> <nucleotide> | <intron> <dinucleotide> | (NBC 6.2.2) <nucleotide><intron> | <dinucleotide> <intron> | <nucleotide> <intron> <nucleotide> | <intron> 5.6. <intron>::= <sequence><domainV><sequence> (NBC 6.3.2) 5.7. <domainV>::= RAGCYNNRURMrNNrAAANNYKYayGYNNRGUUY (NBC 6.3.4) 6. Introns 6.1. 140 <= | *Group I intron* | <= 2,500 (NBC 6.3.1) 6.2. 389 <= | *Group II intron* | <= 3,400 (NBC 6.4.2) 7. D-arm (in addition to CompReq 3) 7.1. 16 <= | *D-arm (with intron)* | <= 3,519 (CR 3.1, NCR 6.1)
**Computational model**
Partial set of problems
Need to re-work completely the analytical approach as previous approach is no longer feasible.

**Figure S2:**
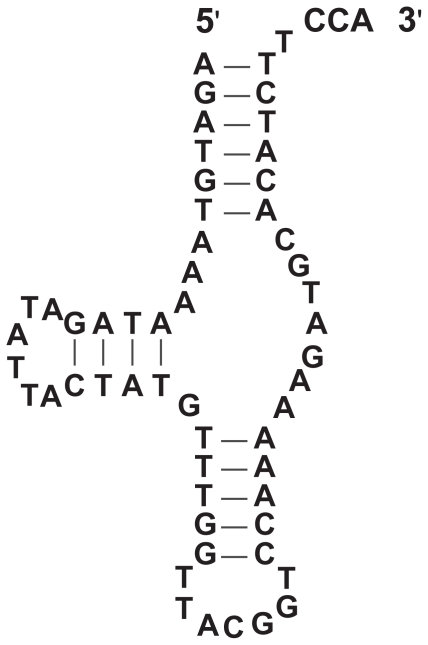
The two-arm, tRNA-Arg molecule in *Caenorhabditis elegans* (Okimoto and Wolstenholme, 1990).

**Figure S3:**
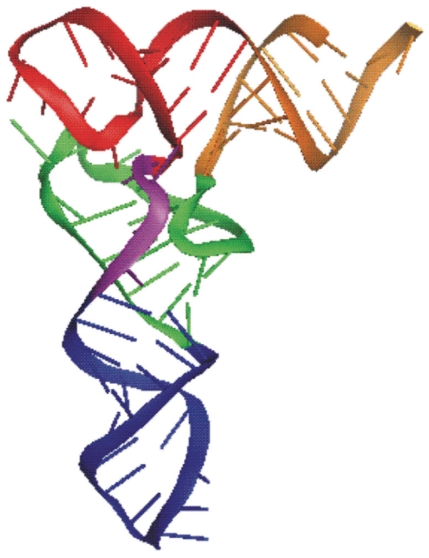
The L-shaped tRNA tertiary structure. AA-stem (orange), D-arm (green), AC-arm (blue), V-loop (purple), T-arm (red).

ReferencesAlbertsBBrayDLewisJRaffMRobertsKWatsonJD1994Molecular biology of the cellGarland Publishing, IncNew YorkBonenLVogelJ2001The ins and outs of group II intronsTrends Genet17322311137779410.1016/s0168-9525(01)02324-1BullerwellCEGrayMW2005*In vitro* characterization of a tRNA editing activity in the mitochondria of *Spizellomyces punctatus*, a Chytridiomycete fungusJ. Biol. Chem2802463701554685910.1074/jbc.M411273200Evguenieva-HackenbergE2005Bacterial ribosomal RNA in piecesMol. Microbiol57318251597806710.1111/j.1365-2958.2005.04662.xLoweTMEddySR1997tRNAscan-SE: a program for improved detection of transfer RNA genes in genomic sequenceNucleic Acids Res2595564902310410.1093/nar/25.5.955PMC146525MarckCGrosjeanH2002tRNomics: analysis of tRNA genes from 50 genomes of Eukarya, Archaea, and Bacteria reveals anticodon-sparing strategies and domain-specific featuresRNA811892321240346110.1017/s1355838202022021PMC1370332OkimotoRWolstenholmeDR1990A set of tRNAs that lack either the T psi C arm or the dihydrouridine arm: towards a minimal tRNA adaptorEMBO J9340511220955010.1002/j.1460-2075.1990.tb07542.xPMC552080PauleMRWhiteRJ2000Survey and summary: transcription by RNA polymerases I and IIINucleic Acids Res281283981068492210.1093/nar/28.6.1283PMC111039SharpSDeFrancoDDingermannTFarrellPSollD1981Internal control regions for transcription of eukaryotic tRNA genesProc. Natl. Acad. Sci. U.S.A78665761694724510.1073/pnas.78.11.6657PMC349108SprinzlMHornCBrownMIoudovitchASteinbergS1998Compilation of tRNA sequences and sequences of tRNA genesNucleic Acids Res2614853939982010.1093/nar/26.1.148PMC147216

## Figures and Tables

**Table 1:** Methodology for formulating a bioinformatics model.

**Biological model**
Biological question
Biological knowledge
Describe biological phenomenon based on observed occurrences
*Basic ‘textbook’ definition and typical scenario*
*Extent and frequency of biological diversity*
Construct dataset of observed instances
*Occurrences representative of the full range of deviations*
*Sample of frequent occurrences*
*All rare and unique instances*
Unobserved yet conceivable occurrences
*Biological structures and mechanisms*
*Knowledge from other systems and other disciplines*
**Bioinformatics transformation**
Biological criteria (BCs)
Convert description of biological knowledge
*Concise, verbal statements for each characteristic*
*Explicit link from each BC into the biological knowledge description*
Remove ambiguity
*Addition or enhancement of BCs*
Computational rules (CRs)
Formulate rules based on BCs
*Mathematical formulas*
*Explicit links to contributing BCs*
Identify key rules
*CRs crucial to the biological phenomenon, the biological question and the computational analysis*
**Computational model**
Global computational problem restating the biological question
General computational approach
Detail approach through a set of smaller problem definitions
